# Assessing the Phytochemical Composition and Effect on Oxidative Stress and Inflammation Molecular Markers of *Celtis integrifolia* extracts in Monosodium Glutamate‐Induced Neurodegenerative Diseases in Mice

**DOI:** 10.1002/alz70859_101708

**Published:** 2025-12-25

**Authors:** Bih Belta Lilian Fubi

**Affiliations:** ^1^ University of Bamenda, Bamenda, Northwest Cameroon

## Abstract

**Background:**

*Celtis integrifolia* called “Ganki‐Djiho” in Hausa, is commonly found in Northern parts of Cameroon. The roots are used in traditional medicine to alleviate nervous problems. The current study sought to assess the neuroprotective potential of *C. integrifolia* against Monosodium glutamate (MSG)‐induced memory deficit in mice model of AD.

**Method:**

Aqueous and ethanol extracts of *C. integrifolia* were investigated for its phytochemicals and cytotoxicity studies (MTT and Hemolysis tests). In different groups of mice (N=5), the extracts were also studied against the effect of MSG in mice. The behavioural paradigms (Y‐maze, Open field test (OFT) and Beam walking test) were performed to evaluate spatial and working memory, exploratory and motor coordination. The biochemical parameters, superoxide dismutase (SOD), reduced glutathione (GSH), malonaldehyde (MDA), catalase (CAT), and nitric oxide (NO) level as hallmarks of oxidative stress were measured. Additionally, the proinflammatory parameters were also determined to evaluate the neuroinflammatory responses associated with MSG such as tumor necrosis factor‐alpha (TNF‐α) interleukin‐1β (IL‐1β), interleukin (IL‐ 6), and interleukin (IL‐10).

**Results:**

Findings obtained revealed that different doses of aqueous and ethanol extracts of *C. integrifolia* significantly reversed the adverse effect of MSG on memory. This was evidence by a significant (*p*<0.001) increment in the percentage of spontaneous alternation in the Y‐maze test and a significant (*p*<0.001) increase in the total distance and number of lines crossed in OFT. Moreover, *C. integrifolia* aqueous and ethanol extracts at different doses caused a significant alteration in biochemical parameters, evidence by significant (*p*<0.001) improvement in antioxidant enzymes activity (CAT, SOD and GSH) associated with a significant (*p*<0.001) decrement in NO and MDA concentrations in brain. Furthermore the extracts significantly (*p*<0.001) decreased the serum neuro‐inflammatory cytokines (IL‐1β, IL‐6 and TNF‐α levels).

**Conclusion:**

The present study attributed the neuroprotective efficacy of *C. integrifolia* in experimental animal model by subsiding the several biomarkers of oxidative stress and neuro‐inflammation.

